# Development of a Rapid and Sensitive Colorimetric Loop-Mediated Isothermal Amplification Assay: A Novel Technology for the Detection of *Coxiella burnetii* From Minimally Processed Clinical Samples

**DOI:** 10.3389/fcimb.2020.00127

**Published:** 2020-05-05

**Authors:** Nazish Sheikh, Sanjay Kumar, Harsh Kumar Sharma, Sameer S. Bhagyawant, Duraipandian Thavaselvam

**Affiliations:** ^1^Biodetector Development Test and Evaluation Division, Defence Research and Development Establishment, Gwalior, India; ^2^Division of Veterinary Public Health and Epidemiology, Sher-e-Kashmir University of Agricultural Sciences and Technology (SKUAST), Jammu, India; ^3^SOS in Biotechnology, Jiwaji University, Gwalior, India

**Keywords:** colorimetric LAMP, qPCR, POC, sensitive detection, *Coxiella burnetii*, Q fever

## Abstract

Q fever is an important zoonotic disease caused by the bacterium *Coxiella burnetii*. The agent is considered as a potential agent for bioterrorism because of its low infectious dose, aerial route of transmission, resistance to drying, and many commonly used disinfectants. Humans are largely infected by the inhalation of aerosols that are contaminated with parturition products of infected animals as well as by the consumption of unpasteurized milk products. Thus, rapid and accurate detection of *C. burnetii* in shedders, especially those that are asymptomatic, is important for early warning, which allows controlling its spread among animals and animal-to-human transmission. In the present study, a colorimetric loop-mediated isothermal amplification (LAMP) assay was developed to confirm the presence of *IS*1111a gene of *C. burnetii* in sheep vaginal swabs. The sensitivity of this assay was found to be very comparable to the quantitative PCR (qPCR) assay, which could detect three copies of the gene, which corresponds to a single cell of *C. burnetii*. The applicability of the colorimetric LAMP assay in the disease diagnosis was assessed by evaluating 145 vaginal swab samples collected from the sheep breeding farms with a history of stillbirth and repeated abortions. Compared to qPCR, colorimetric LAMP had a sensitivity of 93.75% (CI, 69.77–99.84%) and specificity of 100% (CI, 97.20–100%), with a positive (PPV) and negative predictive value (NPV) of 100 and 99.24%, respectively. A very high level of agreement was observed between both colorimetric LAMP and reference qPCR assay. The colorimetric LAMP assay reported here is a rapid and simple test without extensive sample preparation and has a short turnaround time of <45 min. To the best of our understanding, it is the very first study describing the use of colorimetric LAMP assay that detects *C. burnetii* in vaginal swab samples with minimal sample processing for DNA extraction.

## Introduction

Q fever is an important worldwide zoonosis caused by *Coxiella burnetii*, an obligate intracellular bacterium that has a wide host range including humans, ruminants, companion animals, birds, reptiles, and ticks (Angelakis and Raoult, 2010). In human infection, 60% of cases are reported to be asymptomatic. In symptomatic patients, an acute onset is characterized by headache, fever, myalgia, muscle cramps, and frequently an atypical pneumonia or hepatitis. Chronic manifestations may include endocarditis (Maurin and Raoult, 1999). *C. burnetii-*infected animals usually go unnoticed, while certain complications such as abortions, infertility, stillbirth, and metritis are frequently reported. *C. burnetii* shedding is mainly observed during parturition; however, other shedding routes such as milk, vaginal mucus, urine, and feces have also been reported (Porter et al., 2011). Humans are largely infected by the inhalation of aerosols that are contaminated with parturition products of animals as well as by the consumption of unpasteurized milk products (Raoult, 1996; Bouvery et al., 2003). Hence, rapid and accurate detection of *C. burnetii* in shedders is important for early warning, which allows controlling its spread among animals and animal-to-human transmission to obviate devastating outbreaks, such as of Netherlands (2007–2010). The outbreak involved thousands of registered cases of humans along with high reporting of abortions due to Q fever in livestock and accompanied an estimated total cost of 307 million for controlling it, which resulted in huge economic losses to both veterinary industry and society at large (Roest et al., 2011; Van Asseldonk et al., 2013).

*C. burnetii* isolation is time consuming, hazardous, and restricted to BSL-3 laboratories. The serological diagnosis fails to diagnose early infection, as *C. burnetii*-specific antibodies appear only after 2–3 weeks of infection (Fournier et al., 1998). Moreover, these techniques do not demonstrate the current shedding animals (Maurin and Raoult, 1999). Since molecular techniques have been proven to provide rapid and early diagnosis, conventional PCR (Stein and Raoult, 1992; Willems et al., 1994; Ibrahim et al., 1997; Lorenz et al., 1998), nested PCR (Willems et al., 1993; Kato et al., 1998; Zhang et al., 1998a,b), and quantitative PCR (qPCR) (Klee et al., 2006; De Bruin et al., 2013) are used to diagnose *C. burnetii* DNA in different clinical matrices. However, these techniques limits its adoption in resource-poor regions due to the requirement of maintenance of cold chain transportation and need for expensive instrumentation and trained personnel, which preclude the use of PCR at point of care (POC).

Loop-mediated isothermal amplification (LAMP) is a relatively novel type of DNA amplification assay that offers very sensitive, simple, and less time-consuming diagnostic method occurring at temperatures between 55 and 65°C. This assay makes use of a strand displacement activity enabled DNA polymerase and a set of four to six specific primers that recognize six to eight specific target regions, which eliminates the chance of non-specific binding and hence increases the specificity of the assay (Notomi et al., 2000; Nagamine et al., 2002). LAMP is capable of amplifying up to 10^9^ copies of a target in <1 h using simple incubators such as heating blocks or water baths, which makes the method suitable for field conditions and in resource-poor laboratories (Notomi et al., 2000). Moreover, it is reported that LAMP reagents are thermostable at a storage temperature of 25 or 37°C. This, together with resistance of the LAMP assay to inhibitors found in crude clinical samples, makes it amenable to use at POC (Thekisoe et al., 2009). The results of the LAMP assay can be analyzed by observing turbidity with expensive turbidimeter, which restricts its use in resource-poor region (Parida et al., 2005; Zhang et al., 2014). Moreover, results can also be analyzed by performing the agarose gel electrophoresis or addition of intercalating dyes such as SYBR green or propidium iodide postamplification, which increases the risk of amplicon carryover contamination (Parida et al., 2005; Goto et al., 2009). The use of hydroxy naphthol blue (HNB) allows observation of LAMP products with the naked eye, thereby reducing the requirement of instruments. Furthermore adding HNB before the reaction in reaction mix minimizes the risk of obtaining false positives through aerosol, thus enabling the assay to be used in field settings (Goto et al., 2009). Addition of HNB to the reaction produced a visual color change from violet to blue (as the Mg^2+^ ions in solution were chelated by pyrophosphate ions). A positive reaction is indicated by a color change from violet to sky blue (Goto et al., 2009).

The present study describes the development of a rapid, specific, and sensitive colorimetric LAMP assay for the detection of *C. burnetii*. The utility of the assay was applied to sheep vaginal swabs that had undergone a rapid DNA extraction protocol (boiling) and has a short turnaround time of <45 min.

## Materials and Methods

### Design of Colorimetric LAMP Primers

The primers used for colorimetric LAMP assay were designed based on the transposase gene insertion element *IS*1111a (GenBank, accession no. AE016828.2) of *C. burnetii*. Five sets of primers were designed using Primer Explorer V5 software (Table S1). All primers were synthesized by Eurofins MWG Operon (Bangalore, India). The F3 and B3 primers used for the colorimetric LAMP were also made use of to determine the sensitivity of qPCR and conventional PCR.

### *IS*1111a Recombinant Plasmid Construction

To determine the analytical sensitivity of the colorimetric LAMP assay, a plasmid construct was made with the target sequence of *IS*1111a gene (AE016828.2). The region between the F3 and B3 primer was amplified using primers F3 (5′- GACGGGTTAAGCGTGCTC-3′) and B3 (5′- CTGCGCATCGTTACGATCA-3′). The resultant amplicon (194 bp) was cloned into the pGEM-T easy Vector System I (Promega, WI, United States). The recombinant plasmid was quantified by a Nanodrop 1000 (Thermo Scientific, United States) and was diluted serially 10-fold to analyze the sensitivity of the colorimetric LAMP.

### DNA Extraction for qPCR and Colorimetric LAMP

A total of 145 vaginal swab samples were collected from the sheep breeding farms with stillbirth and repeated abortion of Jammu province of Jammu and Kashmir state, India. These samples were initially analyzed by nested PCR as per the protocol of Fenollar et al. (2004), and out of 145 samples, 15 samples were found positive for *C. burnetii*. Crude DNA preparation was performed by firmly pressing the collected vaginal swab against the side of a microfuge tube containing 400 μl of sterile phosphate buffered saline (pH 7.8). A 200-μl saline suspension was heated at 95°C for 5 min and subsequently centrifuged at 10,000 rpm for 5 min to obtain clear liquid on the top (Berri et al., 2001). A 5-μl aliquot of lysis supernatant was used for colorimetric LAMP testing. Total DNA was extracted from the remaining 200-μl saline suspensions left from crude DNA preparation procedure by a DNeasy Blood and Tissue kit (Qiagen) as per the manufacturer's protocols. The resultant 200 μl eluted genomic extract was aliquoted and stored at −80°C until required for qPCR and colorimetric LAMP analysis.

Bacterial genomic DNA for specificity evaluation was extracted using the same DNA extraction kit mentioned above.

### Colorimetric LAMP Reaction

The colorimetric LAMP reaction mixture consisted of 25 μl of total reaction volume, which contained 1 × isothermal amplification buffer (New England BioLabs, MA, United States), 0.8 μM of each of the loop forward (LF) and loop backward (LB) primer, 0.2 μM each of the F3 and B3 primers, and 1.6 μM each of the forward inner primer (FIP) and backward inner primer (BIP), 1.4 mM of deoxyribonucleotide triphosphates (dNTPs) (10 mM each; New England BioLabs, MA, United States), 6 mM MgSO_4_ (100 mM; New England BioLabs, MA, United States), 5 μl of extracted DNA (crude DNA preparation and commercial kit extracted), 8 U of *Bst* Warm Start DNA Polymerase (New England BioLabs, MA, United States), and 0.8 M Betaine (Sigma). The optimization of optimal reaction temperature was accessed by performing a temperature gradient from 55 to 65°C in dry bath for 30 min, and the reaction was terminated by heating at 80°C for 5 min. The results were analyzed by both electrophoresis (2% agarose gels) and by visual detection, which involved the inclusion of HNB dyes at a final concentration of 120 μM during the reaction setup. A positive reaction is indicated by a color change from violet to sky blue.

Each run contained a positive control with *C. burnetii* DNA and a negative control without DNA template. Three different rooms were used for template preparation, LAMP master mix preparation, and the amplification by LAMP to circumvent any carry-over contamination with amplified products.

### Sensitivity and Specificity of Colorimetric LAMP

For the determination of the limit of detection (LOD) and to analyze the reproducibility of the colorimetric LAMP assay, three independent assays were performed employing 10-fold serial dilutions of *IS*1111a recombinant plasmid to concentrations of 3 × 10^7^ to 3 × 10^−1^ copies/μl made in a suitable buffer (25 mM Tris, pH 8.0). One microliter of these dilutions was added as the template to the final reaction volume of 25 μl, and tubes were incubated as per the optimized conditions mentioned earlier.

Similarly, the limit of detection of conventional PCR assay was also performed thrice. The PCR assay was performed using F3 and B3 LAMP primers, which resulted in 194 bp amplicon of the targeted *IS*1111a gene of *C. burnetii*. The PCR mixture contained 0.2 mM of each dNTP, 1 × PCR buffer, 500 nM each of F3 and B3 primers, 1.5 mM MgCl_2_, 1 U of Taq DNA polymerase (MBI Fermentas, Germany), and 1 μl of template DNA. The reaction volume was adjusted to 20-μl by nuclease-free water. The amplification was carried out in the C1000 thermal cycler (BioRad, United States) with a reaction condition of 95°C for 30 s for initial denaturation, followed by 30 cycles of denaturation at 95°C for 30 s, annealing at 61°C for 60 s, extension at 72°C for 30 s, and final extension at 72°C for 5 min. The endpoint detection of amplified products was done on 2.0% agarose gel by electrophoresis.

The specificity of the colorimetric LAMP assay was confirmed employing the DNAs (10 ng/reaction) of closely related bacteria (*Legionella pneumophila* and *Francisella tularensis*). Based on the sequence of its 16S rRNA, *C. burnetii* is classified into the order Legionellales, with *Legionella* spp. and *Francisella* spp. as nearest phylogenetic neighbors (Drancourt and Raoult, 2005). Other bacteria of BW importance such as *Vibrio cholera* **(02)**, *Brucella abortus* **(06)**, *Brucella melitensis* **(06)**, *Burkholderia pseudomallei* **(02)** and *Burkholderia mallei* **(02)**, *Bacillus anthracis* **(02)**, *Salmonella typhi, Shigella dysenteriae*, and *Escherichia coli* were analyzed as described above. The *C. burnetii* NMI and NMII served as positive control. The DNAs used in the experiment were from clinical isolates and standard type cultures.

### qPCR Detection

The qPCR was employed as the reference molecular detection method. For the comparative analysis of the sensitivities of the colorimetric LAMP assay and qPCR, triplicate assays of nine 10-fold serial dilutions (to concentrations of 3 × 10^7^ to 3 × 10^−1^ copies/μl) of *IS*1111a recombinant plasmid mentioned above were used to obtain the standard curves. The qPCR reaction was performed on ABI StepOne Real-Time PCR system (Applied Biosystems, USA) using 2 × FAST SYBR Green Mastermix (Applied Biosystems, United States), 500 nM of forward primer F3, 500 nM of reverse primer B3, and 1 μl of DNA template for sensitivity determination, whereas for screening of vaginal swab samples, 5 μl of extracted DNA (crude DNA preparation and commercial kit extracted) was used in a total of 20 μl volume. The qPCR amplification condition involved an initial activation at 95°C for 20 s, which was followed by 40 cycles of 95°C for 3 s and 60°C for 30 s. The uniqueness of the amplified product was confirmed by performing a melting curve analysis.

Appropriate positive and negative controls were included for each run. To avoid any carry-over contamination with amplified products, proper precautions were taken as discussed earlier as taken for the colorimetric LAMP assay. The results were interpreted according to the software guidelines from the manufacturer (ABI StepOne apparatus, Applied Biosystems, United States).

### Statistical Analysis

The specificity, clinical sensitivity, positive predictive values (PPV), and negative predictive values (NPV) of the colorimetric LAMP assay was compared to those of the qPCR using MedCalc Statistical Software (http://www.medcalc.org). A sample was considered a true positive if it was identified as qPCR positive. The level of agreement was analyzed between colorimetric LAMP and the reference qPCR assay using the kappa (*k*) coefficient with 95% confidence levels (Cohen, 1960).

## Results

### The Best Primer Set

**Figure 1:**
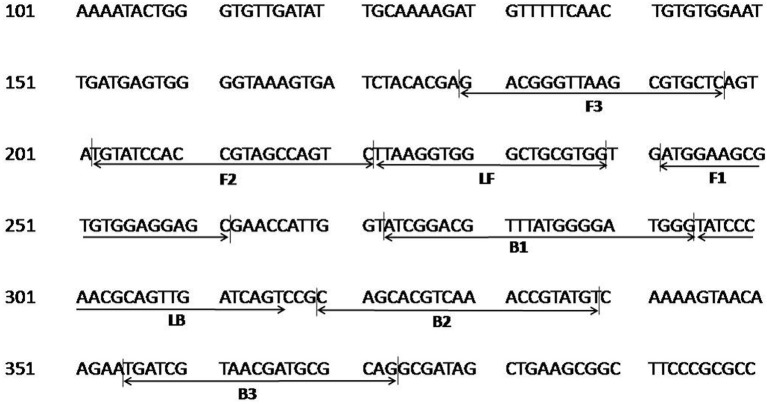
Nucleotide sequence of the transposase gene insertion element *IS*1111a of *C. burnetii* used for designing the LAMP primers. Numbers on the left correspond to the positions in the transposase gene (GenBank, accession no. AE016828.2). The locations of the target sequences are underlined with an arrow. FIP consists of the F1c and F2 sequences, and BIP consists of B1c and B2 sequences.

The colorimetric LAMP assay was carried out with all five primer sets in a range of 55–65°C for 30 min using genomic DNA of *C. burnetii*. The sets 2–5 resulted in inconsistent and non-specific target amplification on repeated runs. The primer set 1 described in Table 1 was found best in amplifying the target consistently at 63°C, and this combination was used for subsequent experiments. The primer FIP, BIP, F3, and B3 has similar sequences to one of the primer set as reported by (Chen and Ching, 2014). The position of primer sequences in the transposase gene insertion element *IS*1111a is depicted in Figure 1.

**Table 1:** List of primer sequences for colorimetric loop-mediated isothermal amplification (LAMP).

**Primer type**	**Sequence (5**′**-3**′**)**
Trans FIP (F1c-F2)	GCTCCTCCACACGCTTCCATTGTATCCACCGTAGCCAGTC
Trans BIP (B1c-B2)	ATCGGACGTTTATGGGGATGGGACATACGGTTTGACGTGCTG
Trans F3	GACGGGTTAAGCGTGCTC
Trans B3	CTGCGCATCGTTACGATCA
Trans LF	CCACGCAGCCCACCTTAA
Trans LB	TATCCCAACGCAGTTGATCAGT

### Colorimetric LAMP Product Detection

**Figure 2:**
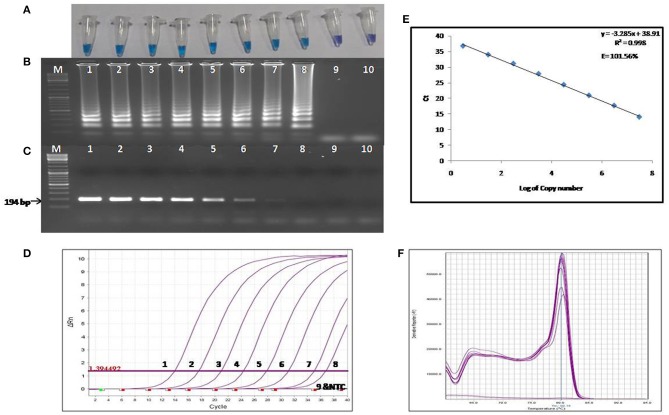
Comparison of the sensitivity of colorimetric LAMP, conventional PCR and qPCR. **(A)** Results of colorimetric LAMP analysis, **(B)** Agarose gel electrophoresis of colorimetric LAMP products, **(C)** Agarose gel electrophoresis of conventional PCR products **(D)** Results of qPCR Analysis, **(E)** Standard curve and amplification efficiency, **(F)** Melting curve analysis. Lane, M: DNA marker; Lanes 1-9: Reaction results from a 10-fold serial dilution of plasmid containing the transposase gene from 3 × 10^7^ to 3 × 10^−1^ copies per reaction; Lane 10: Negative control (nuclease free water).

The colorimetric LAMP products were analyzed by performing agarose gel electrophoresis, and the gel was visualized under UV light post ethidium bromide staining. For the visual detection of the colorimetric LAMP products, HNB dyes were added at a final concentration of 120 μM during the reaction setup. A positive reaction is indicated by a color change from violet to sky blue (Figure 2A).

### Sensitivity of Colorimetric LAMP

The LOD of the colorimetric LAMP assay was determined using the 10-fold serial dilutions of the recombinant plasmid harboring 194 bp amplified fragment of *IS*1111a gene of the *C. burnetii*. The LOD of colorimetric LAMP and conventional PCR assay was found to be 3 and 30 copies/reaction, respectively (Figures 2A–C). Hence, the colorimetric LAMP assay was found to be 10 times more sensitive in detecting C*. burnetii* as compared to conventional PCR.

### Specificity of Colorimetric LAMP

The specificity of the colorimetric LAMP assay was assessed employing the DNA (genomic) of closely related bacteria to *C. burnetii* and other bacteria mentioned in Materials and Methods. The colorimetric LAMP assay was found to be very specific, as no cross-reactivity was seen with any of the bacterial species tested.

### Sensitivity and Specificity of qPCR

The LOD of the reference qPCR was determined by amplifying the 10-fold serial dilutions of the recombinant plasmid harboring the *IS*1111a gene fragment of the *C. burnetii*. The detection limit was found to be three copies per reaction (Figure 2D), which is equivalent to about one cell of *C. burnetii*. The sensitivity of the reference qPCR was found to be similar to that of colorimetric LAMP assay. The standard curve generated by qPCR was linear, generating a coefficient of correlation *R*^2^ = 0.998, a slope of −3.28 with an efficiency of 101.56% (Figure 2E). Melting curve analysis revealed the specificity of primers for the target gene sequence, as all the amplified products showed a uniform melting temperature (*T*_m_) of ~80.15°C (Figure 2F).

### Evaluation of the Colorimetric LAMP Assay With Sheep Vaginal Samples

The diagnostic applicability of the colorimetric LAMP assay was evaluated by testing 145 vaginal swab samples collected from the sheep breeding farms with stillbirth and repeated abortion. Results were compared with the results of the qPCR assay. Fourteen samples were found positive by both colorimetric LAMP and qPCR employing crude DNA preparation and DNA extracted with commercial kit. One sample that was found to be negative by the colorimetric LAMP assay was observed positive by qPCR, and this could be most likely due to a low target level (Ct, 38.12) in the sample. Compared to qPCR, colorimetric LAMP had a sensitivity of 93.75% (CI, 69.77–99.84%) and specificity of 100% (CI, 97.20–100%), with a PPV and NPV of 100 and 99.24%, respectively. A very high level of agreement was found between both colorimetric LAMP and reference qPCR assay. Compared to qPCR, the level of agreement was found to be of 96.2% for colorimetric LAMP (*k* = 0.962; strength of agreement, almost perfect). The outcome of the present study is depicted in Table 2. The colorimetric LAMP assay required a total of 45 min (meantime) from the arrival of a swab in the laboratory to the final result dissemination (10 min crude DNA preparation, 5 min for assay setup, and 30 min for colorimetric LAMP), compared to 1 h, 45 min for conventional LAMP with DNA extraction protocol and 2 h, 30 min for the reference qPCR test.

**Table 2:** Performance of colorimetric loop-mediated isothermal amplification (LAMP) for clinical sheep vaginal swabs (*n* = 145) compared to reference CB qPCR method.

**CB qPCR**	**Colorimetric LAMP**	
	**Positive**	**Negative**
Positive (15)	14	1
Negative (130)	0	130
Sensitivity	93.75%	
Specificity	100%	
PPV	100%	
NPV	99.24%	
Agreement (*k* value)	96.2% (*k* = 0.962, almost perfect agreement)	

## Discussion

The majority of human Q fever cases are related to exposure to domestic ruminants. Preventive measure in controlling Q fever includes a temporary breeding ban, antibiotic treatment, vaccination, safe disposal of placentas and fetuses by burning or burying, and isolation of infected animals from the herd (Van Asseldonk et al., 2013). Finally, when no preventive measures can be applied and if too many contaminated animals are involved, the culling of herds is the ultimate solution. All these control measures lead to a huge economic burden; thus, the diagnosis of animals is of paramount importance to prevent human outbreaks (Brom et al., 2015). The diagnosis of Q fever is very difficult because of its non-specific clinical manifestations (Angelakis and Raoult, 2010). The isolation of the agent is the most reliable diagnosis for Q fever, but it is not routinely practiced because of its poor sensitivity in both cell culture and animal inoculation. This procedure also requires skilled personnel and has to be performed in a containment facility of BSL III level (Maurin and Raoult, 1999). Currently, indirect immunofluorescent antibody assay (IFA) and ELISA are the methods of choice for the serological diagnosis of Q fever (Angelakis and Raoult, 2010). However, these methods are not suitable for early disease reporting because *C. burnetii*-specific antibody appear late as disease progress (Maurin and Raoult, 1999). Moreover, these techniques do not provide the status of current shedding animals (Magouras et al., 2017). The molecular-based techniques such as conventional or quantitative PCRs offer a suitable substitute to culture in demonstrating the presence of *C. burnetii* in clinical samples. Several nested PCR or seminested PCR were developed to detect Q fever with a higher level of specificity and sensitivity (Fournier and Raoult, 2003; Turra et al., 2006). qPCR has several advantages with specific reference to sensitivity, quantification, and control of contamination, but at the same time, it also requires a high-end PCR machine (Panning et al., 2008; Schneeberger et al., 2010). However, these assays limit its use in resource-poor regions due to the requirement of maintenance of cold chain transportation and need for expensive instrumentation and trained personnel, which avert the use of PCR at POC. The LAMP assay is an alternative nucleic acid detection method that has many advantages, such as ease of operation, rapidity, low cost, and modest equipment requirements, making it convenient for use in field conditions (Lucchi et al., 2010; Njiru et al., 2012; Pan et al., 2013).

In the present study, a sensitive and specific colorimetric LAMP assay was developed based on the transposase gene insertion element *IS*1111a of *C. burnetii*. The *IS*1111a gene target was selected because it is a highly conserved gene sequence among the different strains of *C. burnetii*, and the presence of multiple copies in the bacteria leads to a higher sensitivity (Chen and Ching, 2014). The assay could detect as low as three copies per reaction supporting the applicability for the detection of infections with low bacteremia. The colorimetric LAMP assay was further tested for its specificity, employing the genomic DNA of closely related bacteria to *C. burnetii* and other bacteria mentioned in Materials and Methods, and the results indicate no cross-reaction among tested bacteria, confirming that the colorimetric LAMP assay is also highly specific.

The applicability of the colorimetric LAMP assay for the disease diagnosis was assessed by evaluating 145 vaginal swab samples collected from the sheep breeding farms with a history of stillbirth and repeated abortions. The screening of vaginal swabs was executed because the shedding of the agent in the vaginal mucus is higher within 14 days of abortion, and also the handling of swab sample is easy in the field settings (Hansen et al., 2011; Guatteo et al., 2012). The vaginal swab samples were collected after disinfection of the vulva with chlorhexidine solution by authorized veterinarians following standard biosafety procedures. The triple-packed samples were transported from the field to the laboratory in a cool box (4–12°C). The samples were processed for DNA extraction in Biosafety Level 3 laboratory, following which swabs were disposed off postautoclaving (Crews and Gaunt, 2008). To the best of our understanding, it is the very first study describing the use of colorimetric LAMP assay that detects *C. burnetii* in vaginal swab samples with minimal sample processing for DNA extraction. Compared to qPCR, colorimetric LAMP had a sensitivity of 93.75% (CI, 69.77–99.84%) and specificity of 100% (CI, 97.20–100%), with a PPV and NPV of 100 and 99.24%, respectively. A very high level of agreement was observed between both colorimetric LAMP and reference qPCR assay. Only two reports are describing the utility of LAMP assay for the *C. burnetii* detection directly from clinical matrices, namely, abortive products and serum samples of humans and animal origin (Pan et al., 2013; Raele et al., 2015). In their study, DNA extraction was done using commercial kits, which is not only time consuming and tedious but also ill-suited in field conditions. Moreover, in these studies, the LAMP results were analyzed by performing 2% agarose gel electrophoresis followed by its staining with ethidium bromide or by SYBR Green I dye aided visual inspection. In the present study, the colorimetric LAMP was shown to be compatible with a simple heating block and the minimal sample processing (direct detection—no extraction protocol) with sensitivity very comparable to the reference method, qPCR capable of detecting a single cell of *C. burnetii*. The present finding implies that it can be well-suited in areas with limited resources, obviating the dependence on expensive high-end instrumentation and tedious nucleic acid extraction procedures. Additionally, HNB used for the analysis of LAMP products is added before amplification, hence minimizing the risk of aerosol contamination, which results in false positives (Goto et al., 2009). Based on the above attributes, the present colorimetric LAMP technique appears to be a promising substitute to qPCR for detecting *C. burnetii*.

In conclusion, we developed a rapid, simple, and sensitive colorimetric LAMP assay for the detection of *C. burnetii*. The present study enables early detection of the infection in shedding animals, which will allow controlling its spread among animals and animal-to-human transmission. Our present method has not only a short turnaround time but also avoided cross-contamination problem and dependence on expensive equipment, which are desirable characteristics amenable to POC use in resource-limited settings.

## Data Availability Statement

All datasets generated for this study are included in the article/Supplementary Material.

## Author Contributions

SK conceived and designed the experiments. NS and SK performed the experiments. SK, HS, SB, and DT analyzed the data. SK and NS wrote the paper. All authors read and approved the final manuscript.

## Conflict of Interest

The authors declare that the research was conducted in the absence of any commercial or financial relationships that could be construed as a potential conflict of interest.
