# Supplementary material for: Development of a Rapid and Sensitive Colorimetric Loop-Mediated Isothermal Amplification Assay: A Novel Technology for the Detection of Coxiella burnetii From Minimally Processed Clinical Samples
Source: Front Cell Infect Microbiol. 2020 May 5;10:127. doi: 10.3389/fcimb.2020.00127 (PMC7214634; doi:10.3389/fcimb.2020.00127)
Supplement: Supplementary file 1 [file Table_1.docx]

**Table S1. Five sets of LAMP primers for detecting *C. burnetii* detection**

| **Primer type** | **Sequence (5’-3’)** |
| --- | --- |
| Set1-Trans FIP (F1c-F2) | GCTCCTCCACACGCTTCCATTGTATCCACCGTAGCCAGTC |
| Set1-Trans BIP (B1c-B2) | ATCGGACGTTTATGGGGATGGGACATACGGTTTGACGTGCTG |
| Set1-Trans F3 | GACGGGTTAAGCGTGCTC |
| Set1-Trans B3 | CTGCGCATCGTTACGATCA |
| Set1-Trans LF | CCACGCAGCCCACCTTAA |
| Set1-Trans LB | TATCCCAACGCAGTTGATCAGT |
| Set2-Trans FIP (F1c-F2) | GGACTGATCAACTGCGTTGGGAGTGTGGAGGAGCGAACCA |
| Set2-Trans BIP (B1c-B2) | ATCGTAACGATGCGCAGGCGTTACCCTGCACAAACCGC |
| Set2-Trans F3 | CGTAGCCAGTCTTAAGGTGG |
| Set2-Trans B3 | GCGCTTGAACGTCTTGTTG |
| Set2-Trans LF | CCATCCCCATAAACGTCCGATA |
| Set2-Trans LB | ATAGCTGAAGCGGCTTCCCG |
| Set3-Trans FIP (F1c-F2) | GGACTGATCAACTGCGTTGGGAGTGTGGAGGAGCGAACCA |
| Set3-Trans BIP (B1c-B2) | ATCGTAACGATGCGCAGGCGTTACCCTGCACAAACCGC |
| Set3-Trans F3 | CGTAGCCAGTCTTAAGGTGG |
| Set3-Trans B3 | GCGCTTGAACGTCTTGTTG |
| Set3-Trans LF | CCCCATAAACGTCCGATACCAA |
| Set3-Trans LB | ATAGCTGAAGCGGCTTCCC |
| Set4-Trans FIP (F1c-F2) | TTGGCTTTTGCCACCGCTTTTGAATTGTTGAACCGGGACGA |
| Set4-Trans BIP (B1c-B2) | GTACAGAGCATCCCGGGGGTTCACCCACGCTCGCATAA |
| Set4-Trans F3 | CGGATGAAACGGGTGTTGAA |
| Set4-Trans B3 | AACTGCCGGGAACGATGA |
| Set4-Trans LF | CTCCTCGTAATCACCAATCGC |
| Set4-Trans LB | GGGTTATTTAACGGCGCTCTC |
| Set5-Trans FIP (F1c-F2) | TTGGCTTTTGCCACCGCTTTTGAATTGTTGAACCGGGACGA |
| Set5-Trans BIP (B1c-B2) | GTACAGAGCATCCCGGGGGTTCACCCACGCTCGCATAA |
| Set5-Trans F3 | CGGATGAAACGGGTGTTGAA |
| Set5-Trans B3 | AACTGCCGGGAACGATGA |
| Set5-Trans LF | TCCTCCTCGTAATCACCAATCG |
| Set5-Trans LB | TTTAACGGCGCTCTCGGT |
